# Integrated Biological Experiments and Proteomic Analyses of *Nicotiana tabacum* Xylem Sap Revealed the Host Response to Tomato Spotted Wilt Orthotospovirus Infection

**DOI:** 10.3390/ijms252010907

**Published:** 2024-10-10

**Authors:** Hongping Feng, Waiwai Mon, Xiaoxia Su, Yu Li, Shaozhi Zhang, Zhongkai Zhang, Kuanyu Zheng

**Affiliations:** 1 Biotechnology and Germplasm Resources Research Institute, Yunnan Academy of Agricultural Sciences, 2238# Beijing Rd., Panlong District, Kunming 650205, China; hongpingfenghappy@163.com (H.F.); thetmin.wai333@gmail.com (W.M.); sxx919@163.com (X.S.); liziyu19901211@126.com (Y.L.); shaozhizhang1981@hotmail.com (S.Z.); 2 Deputy Director of Microbiology Laboratory, Department of Biotechnology Research, Ministry of Science and Technology, Tansoe Rd., Kyaukse 05151, Myanmar

**Keywords:** tomato spotted wilt orthotospovirus, *Nicotiana tabacum* xylem sap, proteomics, infection

## Abstract

The plant vascular system is not only a transportation system for delivering nutrients but also a highway transport network for spreading viruses. Tomato spotted wilt orthotospovirus (TSWV) is among the most destructive viruses that cause serious losses in economically important crops worldwide. However, there is minimal information about the long-distance movements of TSWV in the host plant vascular system. In this this study, we confirm that TSWV virions are present in the xylem as observed by transmission electron microscopy (TEM). Further, a quantitative proteomic analysis based on label-free methods was conducted to reveal the uniqueness of protein expression in xylem sap during TSWV infection. Thus, this study identified and quantified 3305 proteins in two groups. Furthermore, TSWV infection induced three viral structural proteins, N, Gn and Gc, and 315 host proteins differentially expressed in xylem (163 up-regulated and 152 down-regulated). GO enrichment analysis showed up-regulated proteins significantly enriched in homeostasis, wounding, defense response, and DNA integration terms, while down-regulated proteins significantly enriched in cell wall biogenesis/xyloglucan metabolic process-related terms. KEGG enrichment analysis showed that the differentially expressed proteins (DEPs) were most strongly associated with plant-pathogen interaction, MAPK signaling pathway, and plant hormone signal transduction. Cluster analysis of DEPs function showed the DEPs can be categorized into cell wall metabolism-related proteins, antioxidant proteins, PCD-related proteins, host defense proteins such as receptor-like kinases (RLKs), salicylic acid binding protein (SABP), pathogenesis related proteins (PR), DNA methylation, and proteinase inhibitor (PI). Finally, parallel reaction monitoring (PRM) validated 20 DEPs, demonstrating that the protein abundances were consistent between label-free and PRM data. Finally, 11 genes were selected for RT-qPCR validation of the DEPs and label-free-based proteomic analysis concordant results. Our results contribute to existing knowledge on the complexity of host plant xylem system response to virus infection and provide a basis for further study of the mechanism underlying TSWV long-distance movement in host plant vascular system.

## 1. Introduction

The plant vascular system, mainly composed of the xylem and phloem, serves two essential functions: long-distance transport and mechanical support [1]. Early research suggested that the xylem mainly transports water and dissolved mineral nutrients from the soil to other plant parts. In contrast, the phloem transports and photo-assimilates sugar, amino acids, and organic acids [2,3]. However, recent research reveals that the xylem sap and phloem sap contain significant amounts of functional material, such as hormones, RNA, defense proteins, signal proteins, and transcription factors during responses to abiotic and biotic stresses [4,5,6,7]. This evidence suggests that the plant vascular system is a transportation structure for nutrient delivery and a long-distance communication network system that may coordinate plant development and environmental response [1,8,9]. Plant viruses, which systematically infect host plants, also use the plant vascular system for rapid spread throughout the plant. Although most viruses, such as tobacco mosaic virus (TMV), engage in long-distance movement via the phloem, some viruses, including rice yellow mottle virus (RYMV), turnip mosaic virus (TuMV), and tomato bushy stunt virus (TBSV), etc., move via the xylem [10,11,12,13]. The xylem pit membranes may be the key channels for viruses to load/upload [12]. However, the loading and unloading mechanisms within the xylem and their migration to surrounding cells are poorly understood.

Tomato spotted wilt orthotospovirus (TSWV) is a member of the genus *Orthotospovirus*, family *Tospoviridae*, order *Bunyavirales*. TSWV is -ssRNA with three RNA segments, from large to small, they are called L RNA (8.9 kb), M RNA (4.8 kb), and S RNA (2.9 kb), respectively [14]. TSWV is widely distributed globally, causing serious economic losses in many important solanaceous and compositae crops such as tomato, pepper, tobacco, and lettuce [15,16]. Thrips, especially the Western flower thrip (*Frankliniella occidentalis*), are the main vectors that transmit TSWV persistently in a circulative–propagative way [17]. The particles of TSWV are spherical and wrapped with a host-derived membrane, 80~120 nm in diameter, exceeding the diameter of the plasmodesma (Solanaceae plants plasmodesma diameters is 20~50 nm). Therefore, little is known about the mechanism of TSWV spread in systemic host plants. In order to better understand the mechanism of TSWV long-distance movement, this study examined TSWV virions in the vascular system using transmission electron microscopy (TEM). The results showed that TSWV virions were present in the xylem of the tobacco. Further, proteomic analysis revealed critical insight into the xylem sap response to viral infection and the mechanism of long-distance movement of TSWV.

## 2. Results

### 2.1. TSWV Virions Are Present in the Xylem of Nicotiana tabacum cv. K326

**Figure 1:**
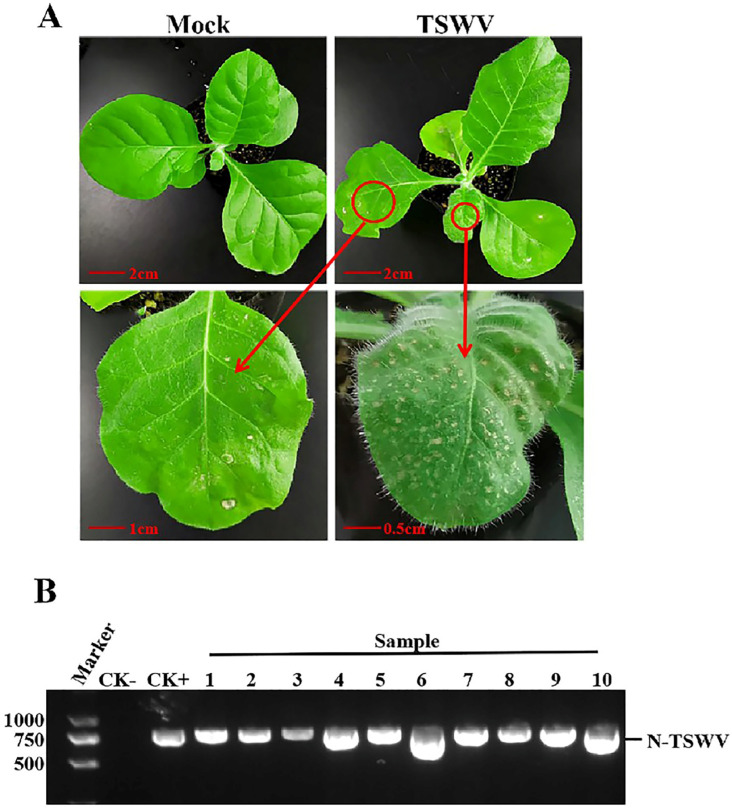
Symptoms of TSWV in *N. tabacum* cv. K326 and RT-PCR detection of TSWV. (**A**) Symptoms of TSWV in *N. tabacum* cv. K326. The red circle and arrow point indicate the symptoms of TSWV infection. Photos were taken 14 days after inoculation. (**B**) RT-PCR identification of TSWV. CK-: healthy tobacco leaf, CK+: positive control infected with TSWV. 1–10: tobacco samples inoculated with TSWV.

**Figure 2:**
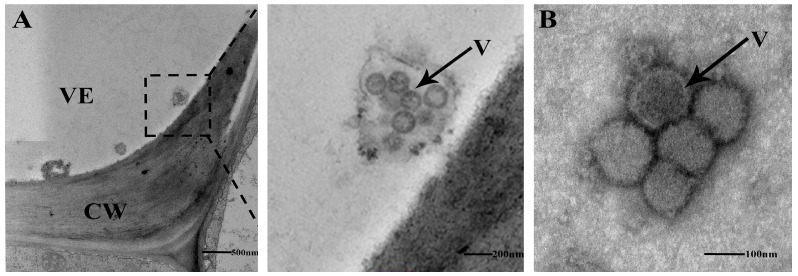
(**A**) The ultrastructure of xylem vessel from TSWV-infected *N. tabacum* cv. K326 was observed by TEM.The dashed box represents the enlarged details of the local area. (**B**) Xylem sap from TSWV-infected *N. tabacum* cv. K326 was collected and observed by TEM following negative staining. VE: vessel, CW: cell wall, V: TSWV virons.

**Figure 3:**
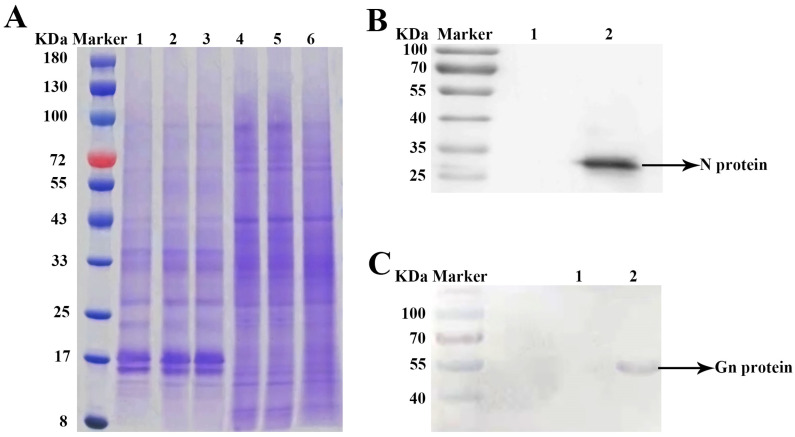
(**A**) Differential proteins between TSWV-infected and mock plants determined via SDS-PAGE analysis. Note: Lane Marker, Lane 1–3: TSWV-infected tobacco xylem sap, Lane 4–6: mock tobacco xylem sap. (**B**,**C**) Western blots of TSWV N protein and TSWV Gn proteins in xylem sap. Note: Lane Marker, Lane 1: mock tobacco xylem sap, Lane 2: TSWV-infected tobacco xylem sap.

Two weeks after inoculation with TSWV, necrosis and shrinkage symptoms appeared on newly opened top leaves of *N. tabacum* cv. K326 (Figure 1A). Systemic infection was confirmed by detecting the N gene of TSWV from newly developed leaves using RT-PCR (Figure 1B). The TEM identified TSWV virions in the xylem (Figure 2A). The TSWV virions were also observed in the collected xylem sap (Figure 2B). Sodium dodecyl sulfate-polyacrylamide gel (SDS-PAGE) and Coomassie blue staining showed significantly different results between TSWV-infected and mock plants (Figure 3A). Western blotting verified TSWV structural proteins, N and Gn, were presented in the xylem sap of the TSWV-infected group (Figure 3B,C).

### 2.2. Multiple Analysis of Differentially Expressed Proteins

#### 2.2.1. Differentially Expressed Proteins

**Figure 4:**
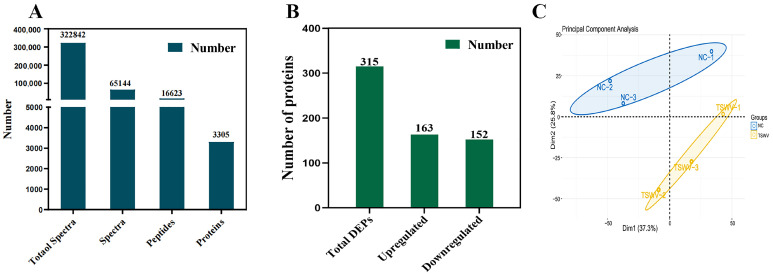
(**A**) Number of xylem sap proteins identified by LC-MS/MS. (**B**) The number of upregulated and downregulated proteins. (**C**) PCA of the tobacco xylem sap samples.

Reverse phase HPLC and LC-MS/MS proteomic generated 322,842 spectra, including 16,623 peptides and 3305 quantified proteins in two groups (Figure 4A). And 315 differentially expressed proteins (DEPs) (*p*-value < 0.05) (163 up-regulated and 152 down-regulated) were identified (Figure 4B). In addition, three TSWV structural proteins, N, Gn and Gc, were identified in the TSWV infection group. Principal component analysis (PCA) revealed two categories: Dim1 (37.3%) (TSWV-1, TSWV-2, and TSWV-3) and Dim2 (25.8%) (NC-1, NC-2, and NC-3), explaining 63.1% of protein changes (Figure 4C).

#### 2.2.2. GO Enrichment and KEGG Pathway Enrichment Using the DAPs in Response to TSWV Infection of the Xylem Sap

**Figure 5:**
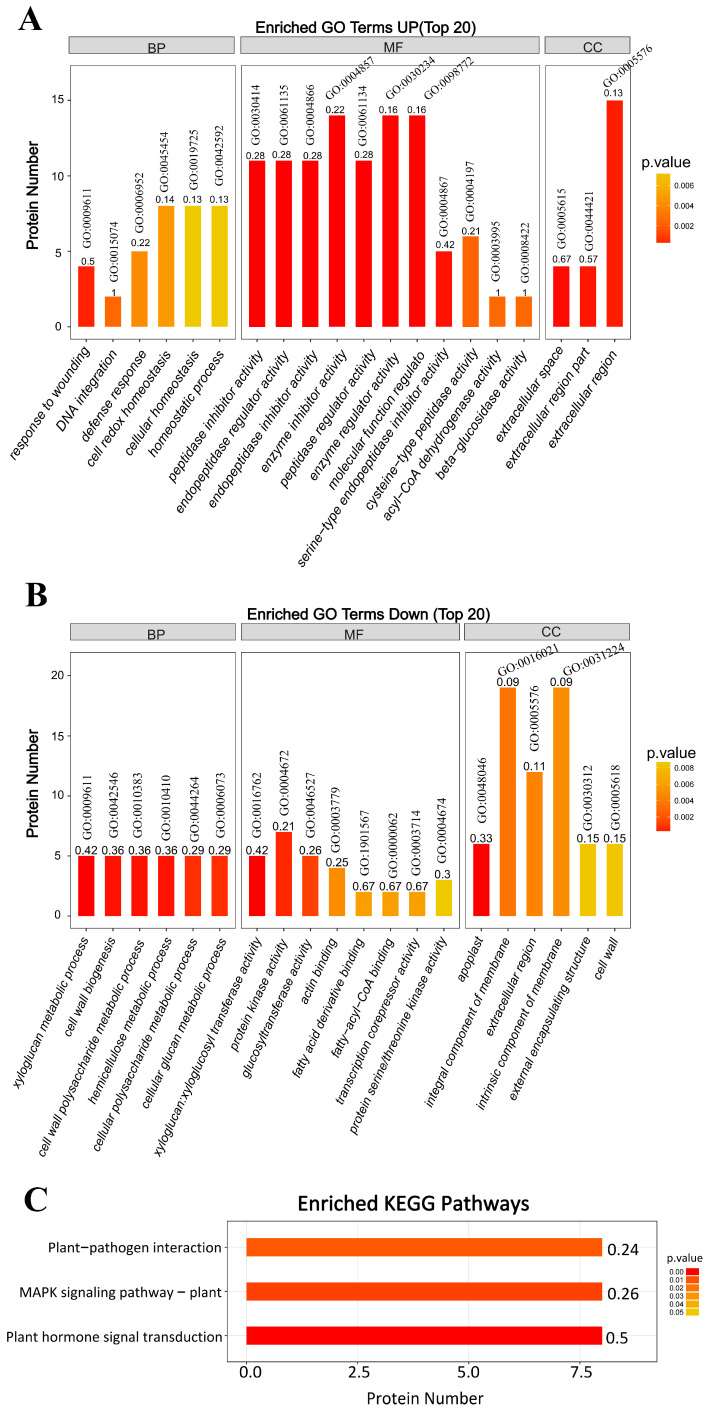
GO enrichment and KEGG pathways. BP: biological process; MF: molecular function; CC: cellular component. (**A**) The upregulated proteins that significantly enriched GO terms. (**B**) The downregulated proteins significantly enriched GO terms. (**C**) KEGG pathway analysis.

The up-regulated proteins significantly enriched in cellular homeostasis (GO:0045454, GO:0042592, GO:0019725), response to wounding (GO:0009611), defense response (GO:0006952), and DNA integration (GO:0015074) under the biological process (BP) category. In the molecular function (MF) category, the proteins significantly enriched several cell-regulation related terms, including GO:0004866, GO:0061135, GO:0030414, GO:0004857, GO:0061134, GO:0030234, GO:0098772, and GO:0004867. The enriched GO terms under the cellular component (CC) category belong to extracellular region/space, including GO:0005615, GO:0044421, and GO:0005576 (Figure 5A). The down-regulated proteins significantly enriched cell wall biogenesis/xyloglucan metabolic process-related terms, including GO:0010411, GO:0010383, GO:0010410, GO:0042546, GO:0006073, and GO:0044264. All the enriched GO terms related to protein ID were annotated to xyloglucan endo-transglucosylase. Under MF, the down-regulated proteins were enriched in xyloglucosyl transferase/glucosyltransferase activity (GO:0016762, GO:0046527), protein kinase activity (GO:0004672, GO:0004674), actin binding (GO:0003779), fatty acid derivative/fatty-acyl-CoA binding (GO:1901567, GO:0000062), and transcription corepressor activity (GO:0003714) (Figure 5B).

Further, KEGG analysis showed that plant–pathogen interaction, MAPK signaling, and plant hormone signal transduction pathways were the most affected (Figure 5C). In the plant–pathogen interaction pathway, TSWV infection significantly down-regulated the proteins associated with hypersensitive response (HR), including calcium-dependent protein kinase (CDPK) and heat shock protein 90A. In the MAPK signaling pathway, TSWV infection significantly up-regulated the pathogenesis-related protein 1 (PR1), acidic endochitinase related to pathogen attack, and abscisic acid receptor PYR1 related to the abscisic acid-induced pathway. In the plant hormone signal transduction pathway, TSWV infection significantly up-regulated four hormone signal transduction pathways: the salicylic acid pathway (the PR1 induced by salicylic acid was significantly upregulated), abscisic acid pathway (the related protein PYR1 was significantly upregulated), cytokinin pathway (the two-component response regulator ARR2 was significantly upregulated), and the brassinosteroid pathway (the brassinosteroid insensitive 1 (BRI1) was upregulated considerably).

#### 2.2.3. Cluster Analysis of Differentially Quantified Proteins

**Figure 6:**
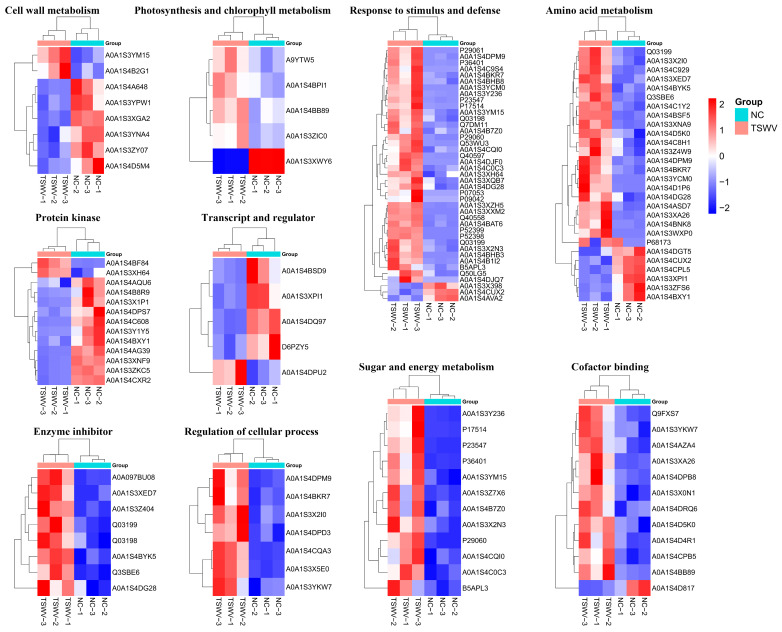
Cluster analysis of DEPs.

According to cluster analysis of DEPs function, the DEPs were involved in cell wall metabolism, sugar and energy metabolism, amino acid metabolism, and stress/defense, etc. (Figure 6, Table S1). In the cell wall metabolism cluster, two up-regulated proteins (xylose isomerase and glucan endo-1,3-beta-D-glucosidase) and seven down-regulated proteins (xylologlucan endo-transglucosylase/hydrolase, polygalacturonase, galactosidase, xylosidase, and fructofuranosidase) were involved. Furthermore, TSWV infection up-regulates enzyme inhibitors, regulates the cellular process, as well as sugar and energy metabolism in xylem sap. lPRemarkably, TSWV infection affects photosynthesis, as a photosynthesis protein (PSI subunit V) was significantly down-regulated. These results indicate a sensitive proteomic response when TSWV infects tobacco.

#### 2.2.4. PPI Network Analysis of DEP Interactions

**Figure 7:**
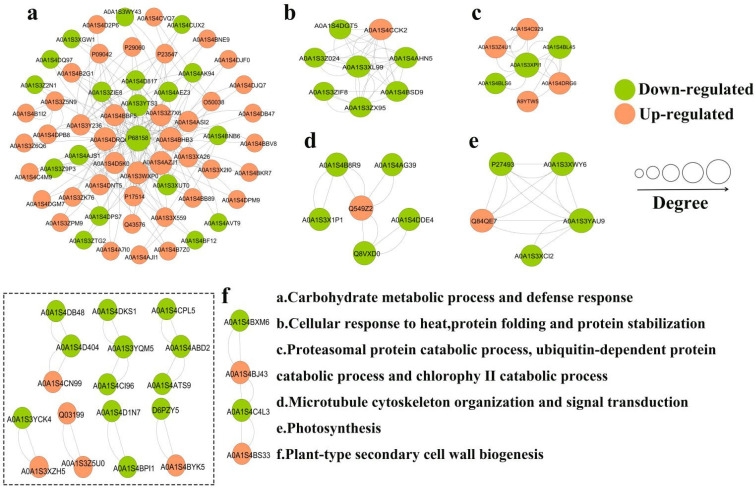
PPI network analysis of DEPs.

A PPI network of DEPs revealed six highly interconnected clusters (Figure 7, Table S2). Cluster a involved carbohydrate metabolic process and defense response, and P68158 (Elongation factor TU) appeared as a key protein in this network. Cluster b involved several heat shock proteins, most of which were down-regulated in the TSWV infection group. Cluster c involved protein catabolism. Proteasome subunit alpha type was significantly up-regulated, while ubiquitin-NEDD8-like protein RUB2 was down-regulated, and NEDD8 ultimate buster 1-like was up-regulated. Cluster d had six proteins (involving microtubule cytoskeleton organization and signal transduction), where one protein (14-3-3 protein) was up-regulated and five proteins were down-regulated. Then, cluster e (photosynthesis) had five proteins, with one protein (photosystem I reaction center subunit III) was up-regulated and four proteins (chlorophyll a-b binding protein, chlorophyll a-b binding protein 21, PSI subunit V and rab escort protein 1) were down-regulated. In addition, other small clusters have also been found, including fatty acid metabolic process, phenylpropanoid metabolic process, and plant-type secondary cell wall biogenesis, etc. (Table S2), suggesting that these small clusters also play an important role in regulatory networks.

### 2.3. PRM Quantification of 20 Candidate Proteins

**Figure 8:**
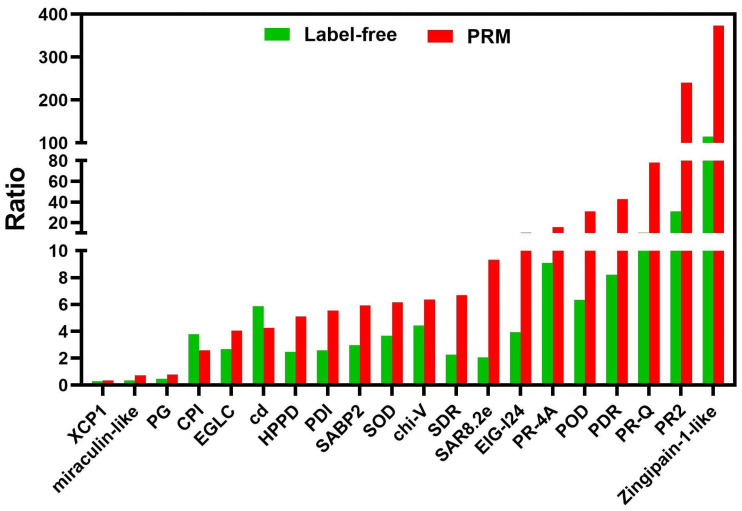
Comparison of the quantification results between label-free and PRM of the 20 candidate proteins. The *X*-axis represents the protein names, and the *Y*-axis represents fold changes of protein abundances between TSWV-infection and control in tobacco xylem sap.

Here, the PRM technique was used to validate the reliability of the 20 candidate DEPs from label-free-based proteomic data (Figure 8, Table S5). Seventeen proteins were up-regulated, and three were down-regulated. All the PRM-detected proteins were consistent with the label-free quantification results. Thus, the label-free results were credible for further analysis involving DEPs with their GO annotations and KEGG pathways, as well as the regulatory networks during TSWV infection of tobacco. We identified the regulatory networks that respond to TSWV in tobacco.

### 2.4. Relative Expression of Candidate Genes Based on DEPs

**Figure 9:**
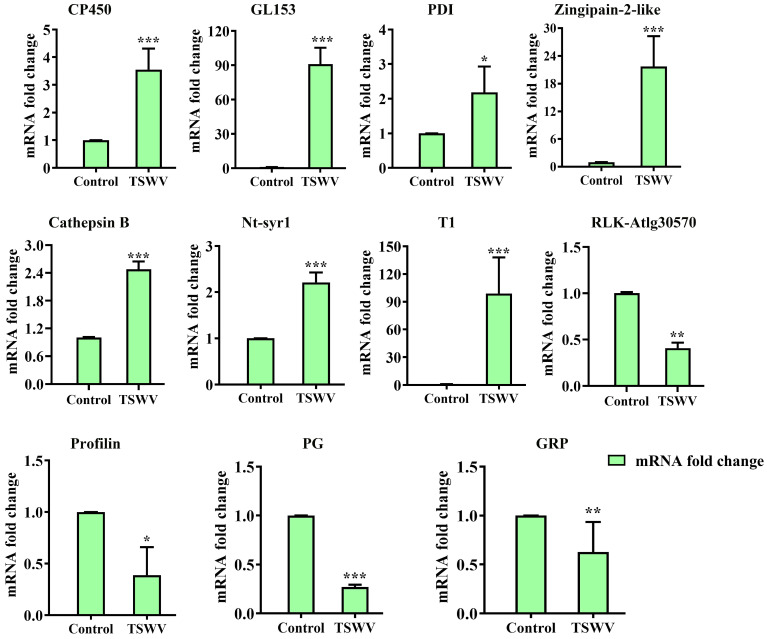
qRT-PCR analysis of 11 candidate genes with significant changes in protein abundance. The bars represent the means ± SD (*n* = 3) of three biological replicates. The asterisks indicate the significance level (* *p* < 0.05, ** *p* < 0.01, *** *p* < 0.001) based on a Student’s *t*-test.

RT-qPCR validation was performed using 11 genes (Table S3) to evaluate the reliability of the DEPs. Table S4 contains the designed gene-specific primers. The 11 proteins are involved in ATP binding and ATP transmembrane transport (probable receptor-like protein kinaseAt1g30570 (RLK-AtIg30570) and probable ADP, ATP carrier proteinAt5g56450 (*CP450*)), stress and defense metabolism (Protein disulfide-isomerase (PDI) and (Trypsin inhibitor 1 (*T1*)), carbohydrate metabolic (Glucan endo-1,3-beta-glucosidase 5-like (*GL153*) and probable polygalacturonase (*PG*)), vesicle docking and vesicle fusion (Syntaxin-related protein Nt-syr1 (*Nt-syr1*)), cytoskeleton (Profilin), proteolysis involved in protein catabolic process (*Zingipain-2-like* and *Cathepsin B*), and nucleic acid binding (glycine-rich protein 2-like (*GRP*)). TSWV infection significantly up-regulated the genes expression of *CP450*, *GL153*, *PDI*, *Zingipain-2-like*, *Cathepsin B*, *Nt-syr1*, and *T1* at 14 days post-inoculation (dpi) while significantly down-regulated the genes expression of *RLK-AtIg30570*, *Profilin*, *PG*, and *GRP* (Figure 9). Thus, the concordant results of the RT-qPCR and label-free-based comparative proteomic analysis further validate the label-free findings.

## 3. Discussion

Tomato spotted wilt orthotospovirus (TSWV) is currently one of the plant viruses with the widest known host range, capable of infecting over 925 species of plants in 70 families ranging from monocotyledonous to dicotyledonous, causing serious harm [18]. Previous studies poorly understood how TSWV moves systematically in host plants, i.e., through phloem or xylem? How to load/unload from TSWV phloem or xylem? Which host factors are involved in systemic movement of TSWV? In this study, The TEM and western blotting results revealed the presence of TSWV virions and structure proteins in tobacco xylem sap, indicating TSWV may engage in long-distance movement via the xylem. This is the first study about TSWV detection in xylem sap. Further, the quantitative proteomic analysis based on label-free methods was conducted to reveal the uniqueness of protein expression in xylem sap during TSWV infection. The results contained 315 differentially expressed proteins (DEPs) (*p*-value < 0.05) (163 up-regulated and 152 down-regulated). Here we discuss the potential functions of these DEPs in major metabolic pathways and investigate the molecular mechanisms of *Nicotiana tabacum* xylem adaption to and defense against TSWV infection.

### 3.1. TSWV Infection Affects Cell Wall Metabolism and Remodeling of Host Pant Tobacco K326

Cell wall-related proteins are central in modulating cell wall extensibility, which mediates cell enlargement and expansion. The xylem of some plants contains cell wall-related proteins, and their functions may involve differentiation and morphogenesis of vessel molecules [2,3]. In this study, the xylem sap from healthy controls had high levels of several cell wall-related proteins, including xylologlucan endo-transglucosylase/hydrolase (XTH), polygalacturonase (PG), galactosidase, xylosidase, and fructofuranosidase, which are involved in cell wall relaxation, elongation, and remodeling. We further investigated the expression of PG by PRM and RT-qPCR, and the results were consistent with the proteomic data. However, TSWV infection significantly down-regulated the expression of these cell wall-related proteins but significantly up-regulated other cell wall-related proteins, such as glucan endo-1,3-beta-glucosidase.

Furthermore, some receptor-like protein kinase (RLK) was highly expressed in healthy controls and significantly repressed in TSWV-infected groups, including some receptor-like protein kinases which are related to cell wall thickening [19,20]. For instance, several receptor-like protein kinases identified in this study, including receptor-like protein kinase, receptor-like protein kinase AT1G30570, and receptor-like protein kinase Feronia [21,22] are related to plant growth and development, seed development, and cell morphogenesis. However, there is no direct evidence to prove their relation to vessel cell wall thickening (Figure 6, Table S1).

### 3.2. Disruption and Re-Regulation of Cell Redox Homeostasis of Host Plant during TSWV Infection

Pathogen infection induces ROS production. However, plants use specific mechanisms to stabilize their intracellular redox balance, regulating the redox imbalance caused by TSWV infection. This study did not detect the level of ROS during TSWV infection. However, TSWV infection significantly up-regulated ROS-related antioxidant proteins that can directly or indirectly eliminate H_2_O_2_. Therefore, TSWV infection induced excess ROS and oxidative stress in host plants. This study showed that intracellular redox balance might involve many different types of functional proteins, including: 1. peroxidases with direct antioxidant activity, such as superoxide dismutase (SOD); 2. glutathione reductase and glutathione transferase; 3. dehydrogenase; 4. Thioredoxin (Table S1), based on the reason of these proteins were significantly up-regulated in the TSWV infected group.

### 3.3. TSWV Infection-Induced PCD Pathway

In eukaryotes, genes that participate in plant growth and development, biological/abiotic stress, and other processes regulate programmed cell death (PCD), an active death mode. Various signal molecules such as ROS, Ca^2+^, ethylene, and salicylic acid induce PCD in plants. Moreover, the release of cytochrome c and cytochrome b5 from mitochondria into the cytoplasm and the activation of vacuolar processing enzymes (VPE), metacasase, saspases, and other animal-like caspases are the key steps to PCD response. In this study, the xylem sap of TSWV-infected host plants had PCD signal cytochrome c, which were lacking in healthy control plants. Additionally, the xylem sap of TSWV-infected plants had 20S proteasome and cathepsin B, the marker proteins of plant PCD response with animal caspase-3-like activity [23,24]. In addition, TSWV infection significantly up-regulated several cysteine-type endopeptidases, such as zingipain-2-like and senescence-specific cysteine protease SAG12-like (Figure 8 and Figure 9, Table S1). These results indicate that TSWV infection induces a PCD response, and the relevant signal molecules may be transported to the upper part of the leaf through the xylem, thereby causing the PCD response in that part.

### 3.4. Host Defense Response to TSWV Infection

#### 3.4.1. Receptor-like Kinases (RLKs)

Receptor-like kinases are one of the most abundant classes of plant proteins. They perceive and transduce signals from the extracellular environment into cells. Plant RLKs are involved in nearly all aspects of plant life, including growth, development, and stress response [25].

In this study, the xylem sap of TSWV-infected plants had significantly up-regulated levels of two RLKs: somatic embryogenesis receptor kinase 2-like isoform 2 (SERK2), and Cysteine-rich receptor-like protein kinase 10 (CRK10). These two RLKs belong to different RLKs subfamilies; SERK2 belongs to the somatic embryogenesis receptor-like kinase (SERKs) subfamily, a leucine-rich receptor kinase (LRR-RLKs). The SERK members include SERK1, SERK2, BAK1/SERK3, BKK1/SERK4, and SERK5, and are key in brassinolide (BR) signal transduction, cell death regulation, and pathogen-mediated innate immunity [26,27,28]. SERK2 has been associated with pathogen- or danger-associated molecular patterns (PAMP/DAMP) and signaling [29,30]. Furthermore, SERK2 is also a BR signaling component, which plays a vital role in anti-biotic and abiotic stress [27,31,32] and plant growth and differentiation [33,34].

Cysteine-rich receptor-like protein kinase 10 belongs to the oysteine-rich repeat RLKs (CRKs) subfamily. Many CRK-encoding genes are involved in ROS/redox signaling and sensing [35], activation of hypersensitive cell death during pathogen infection [36], enhanced pattern-triggered immunity (PTI), and regulation of PAMP/DAMP [37,38].

#### 3.4.2. Pathogenesis Related (PR) Protein

Pathogens or chemicals (such as S, H_2_O_2_) induce PR proteins expression, which play an important role in disease resistance, responding to biological or abiotic stress, and adapting to adverse environments. The PR proteins belong to 17 families [39] in different plants. In this study, TSWV infection significantly up-regulated twenty-four PR proteins in the xylem sap (Table S1). Among them, PR4 and PR10 have ribonuclear RNase and DNase activity, thus directly playing an antiviral function [40,41,42]. PR4 and PR10 are responsible for cell death-mediated defense signaling [43,44]. However, virus infection induces the other PR proteins, such as PR1, PR2, and PR3 [45,46,47], that mainly function against pathogenic fungi and bacteria, with no direct antiviral activity [48,49]. Therefore, their antiviral effect probably functions indirectly and still needs further study.

#### 3.4.3. Salicylic Acid Binding Protein (SABP)

Salicylic acid (SA) is a key plant hormone that mediates host defense responses to biotic stress in plants. Accordingly, SABP is among the key enzymes that activate the SA-mediated defense pathway [50]. Tobacco has three SABP kinds, named SABP-1, SABP-2, and SABP-3 [51,52]. SABP-2 can bind to SA with high affinity and has the methylesterase activity that catalyzes the conversion of methyl salicylate (MeSA) into SA, which is necessary to induce systematic acquired resistance (SAR) in plants [53]. In this study, TSWV infection significantly up-regulated SABP-2 in the xylem sap, which was confirmed by both label-free and PRM methods. Silencing SABP-2 can inhibit the expression of PR1 and reduce the resistance to TMV [54]. This study suggests that upward SABP transportation through the xylem may be key to obtaining SA-mediated SAR in the developing upper leaves.

#### 3.4.4. DNA Methylation

DNA methylation is an important form of epigenetic modification, which plays an essential role in regulating plant growth and development and responding to abiotic stresses. In this study, TSWV infection up-regulated three proteins (putative methyltransferase DDB_G0268948, serine hydroxymethyltransferase (SHMT), and adenosylhomoysteinase (SAHase)) that are related to methyl transfer or methyl cycle in the xylem. It was found that C1 protein encoded by geminnivirus can intertact with SAHH and reduce SAHH activity, thus repressing methylation during virus infection [55].

#### 3.4.5. Proteinase Inhibitor (PI)

Proteinase inhibitors (PIs) are important in plant disease resistance to biological stress. In this study, TSWV infection significantly up-regulated eight protease inhibitors in the xylem (Table S1). These eight proteases inhibitor include a serine proteinase inhibitor, cysteine proteinase inhibitor, trypsin proteinase inhibitor, and metalloproteinase inhibitor. The functions of PIs mainly include two aspects: insect resistance and inhibiting pathogen growth. Serine protease inhibitors inhibit most lepidopterans, orthoptera, diptera, hymenoptera, and some coleoptera because serine proteases are the major digestive enzymes in these insects.

Meanwhile, cysteine protease inhibitors mainly inhibit coleoptera insects because cysteine protease is the main digestive enzyme of coleoptera [56]. Proteinase inhibitors also directly inhibit pathogen growth, thus improving plant disease resistance [57,58]. The current evidence of PIs’ antiviral activity include: TMV infection can rapidly induce protease inhibitors [59]; tobacco plants over-expressing the rice cysteine protease inhibitor show resistance to tobacco etch virus (TEV) and potato virus Y (PVY) [60]; and the miraculin-like protein hijacks the viral movement-related p33 protein and induces cellular oxidative stress in defense against the citrus tristeza virus (CTV) [61]. The high expression of PI in the host plant during TSWV infection may inhibit the feeding of thrips or have a direct anti-TSWV function [62].

## 4. Materials and Methods

### 4.1. Plant Materials and Virus Inoculation

The *Nicotiana tabacum* cv. K326 plants were grown in an illumination incubator at 25 °C with a 16/8-h (light/dark) photoperiod. At approximately 4–6 weeks, TSWV isolate 14YV283 was maintained in *Nicotiana tabacum* by mechanical inoculation using 0.05 M sodium phosphate buffer containing 0.01 M sodium sulfite, pH 7.0. Thirty K326 plants were in a group, with three replications. The control group consisted of healthy plants and was only inoculated with buffer solution. All plants were cultivated at 25 °C in a temperature-controlled greenhouse. The xylem sap was collected 14 days post inoculation (dpi) [63].

### 4.2. RT-PCR Using for TSWV Detection

A total of 0.2 g of tobacco leaf was utilized for the extraction of total RNA.Total RNA was extracted using the RNAiso Plus kit (TaKaRa Bio, Shiga, Japan), and first-strand cDNA synthesis was performed with the PrimeScript RT Reagent Kit (Takara, Dalian, China). Amplification of the fragments corresponding to the nucleoprotein gene of TSWV was carried out utilizing Primer STAR Max^®^ DNA Polymerase (Takara, Dalian, China) with the prepared cDNA as template material. The specific primers for TSWV-N gene are “TSWV-N-F: 5′-ATGTCTAAGGTTAAGCTCACT-3′; TSWV-N-R: 5′-TTAAGCAAGTTCTGCAAGTT-3′”. The purified PCR products were cloned into the pMD18-T vector (Takara, Dalian, China) and subjected to sequencing analysis by Shanghai Biotechnology and Biological Engineering (Shanghai, China) Technology Co., Ltd.

### 4.3. TEM Observation

The inoculated samples on 14 dpi were used for the ultrathin section and TEM observation. Stem tissues of K326 tobacco were cut into small pieces of 1 mm × 3 mm and fixed with 2.5% glutaraldehyde solution. Then, the samples were dehydrated with 30%, 50%, 70%, 80%, 90%, 95%, and 100% ethanol for 15 min at each stage. After another 20 min of dehydration with 100% ethanol, the samples were dehydrated with acetone for 20 min. The dehydrated sample was immersed overnight in pure embedding agent Epson-812, then samples were put into a polymerization box to polymerize the sample twice at 70 °C for 12 h or 60 °C for 24 h. The polymerized sample was cut into 70~90 nm sections by Leica-UC7 microtome (LEICA, Wetzlar, Hessian, Germany). Then, first stain with uranyl acetate for 5–10 min, and then stain with lead citrate for 5 min. A TaicnaiG2 Spieit transmission electron microscope (TEM, FEI, Hillsboro, OR, America) was used to observe the prepared sample. For negative staining sample preparation, the copper mesh was placed on the xylem sap and incubated for 3 min. Then, filter paper was used to absorb the liquid on the surface of the copper mesh. Finally, the copper mesh was stained in phosphotungstic acid solution (2% *w*/*v*) for 2 min and observed by TEM.

### 4.4. Xylem Sap Collection

The collection of xylem sap was based on a protocol that was described previously [13]. Briefly, after two weeks, all the TSWV-inoculated plants exhibiting necrosis and leaf shrinkage were used for xylem sap collection while non-inoculated plants were used as controls. The stems were cut into 2 cm long segments, washed three times with sterile water to avoid contamination from injured cells, and dried on filter papers. Next, each of the stems were placed in 1.5 mL centrifugation tubes and centrifuged at 3000 rpm for 10 min at 4 °C to collect xylem sap. A total of 30 plant samples’ xylem sap were mixed as a group and repeated three times in each group. The collected xylem sap was immediately frozen with liquid nitrogen and stored at −80 °C.

### 4.5. Xylem Sap Protein Extraction and Detection

The 10 kDa ultrafiltration tube (Sartorius) was used for concentrating low-abundance components. One volume of SDT buffer (4%SDS, 100 mM Tris-HCl, 1 mM DTT, pH7.6) was added to the sample; the mixture was then boiled for 15 min and centrifuged at 14,000× *g* for 40 min. The supernatant was quantified using the BCA Protein Assay Kit (Bio-Rad, Hercules, CA, USA) and stored at −80 °C. The xylem sap protein was identified using SDS-PAGE stained with Coomassie brilliant blue. Western blotting was performed by wet transfer to observe how the obtained antiserum recognizes the TSWV N and TSWV Gn proteins. Briefly, proteins were transferred to the PVDF membrane and blocked at RT for 1 h with a blocking solution (1 × TBS-Tween 20 buffer [TBST] containing 5% nonfat powdered milk [*w*/*v*]). The antiserum was dissolved in the blocking solution at a dilution factor of 1:3000 and incubated at 4 °C for 12 h. Then, the PVDF membrane was incubated at RT for 1 h. The TSWV N and Gn polyclonal antibody used in this experiment came from Key Laboratory of Plant Immunity, Department of Plant Pathology, Nanjing Agricultural University. The AP-labeled goat anti-rabbit (Invitrogen, Carlsbad, CA, USA) was used as the secondary antibody (dissolved in the blocking solution with dilution factor 1:5000). The target bands were observed with BCIP/NBT Color reagent kit (Coolaber, Beijing, China).

### 4.6. Label-Free Quantitative Proteomics

#### 4.6.1. High-Performance Liquid Chromatography (HPLC) Fractionation and Liquid Chromatography-Mass Spectrometry/Mass Spectrometry (LC-MS/MS) Analysis

Each fraction was injected for nano LC-MS/MS analysis. The peptide mixture was loaded onto a reverse phase trap column (Thermo Scientific Acclaim PepMap100, 100 μm × 2 cm, nanoViper C18) and connected to the C18-reversed phase analytical column (Thermo Scientific Easy Column, 10 cm long, 75 μm inner diameter, 3 μm resin) in buffer A (0.1% Formic acid). The mixture was separated using a linear gradient of buffer B (84% acetonitrile and 0.1% Formic acid) at a 300 nL/min flow rate controlled by IntelliFlow technology. The linear gradient condition was: 35% buffer B for 50 min, 35–100% buffer B for 5 min, and hold in 100% buffer B for 5 min. The LC-MS/MS analysis was performed on a Q Exactive mass spectrometer (Thermo Fisher Scientific, Waltham, MA, USA) coupled to an Easy nLC (ProxeonBiosystems, now Thermo Fisher Scientific, Waltham, MA, USA) for 300 min. The mass spectrometer was operated in a positive ion mode. The MS data was acquired using a data-dependent top10 method, dynamically choosing the most abundant precursor ions from the survey scan (300–1800 *m*/*z*) for HCD fragmentation. The automatic gain control (AGC) target was 3 × 10^−6^, maximum inject time was 10 ms, and the dynamic exclusion duration was 40.0 s. Survey scans were acquired at a resolution of 70,000 at *m*/*z* 200; the resolution for HCD spectra was set to 17,500 at *m*/*z* 200; the isolation width was 2 *m*/*z*. The normalized collision energy was 30 eV, and the underfill ratio, which specifies the minimum percentage of the target value likely to be reached at maximum fill time, was defined as 0.1%. Finally, the instrument was run in the peptide recognition mode.

#### 4.6.2. Database Search and Bioinformatics Analysis

The MS data were analyzed using the MaxQuant software version1.5.3.17 (Max Planck Institute of Biochemistry, Martinsried, Germany). The protein sequences of DEPs were in batches retrieved from the UniProtKB database (Release 2016_10) in FASTA format. The retrieved sequences were locally searched against the SwissProt database (tobacco) using the NCBI BLAST+ client software (ncbi-blast-2.2.28+-win32.exe) to find homologue sequences from which to transfer the functional annotation to the studied sequences. Then, the top 10 blast hits with an E-value < 1 × 10^−3^ for each query sequence were retrieved and loaded into Blast2GO9 (Version 3.3.5) for GO mapping and annotation. The annotation threshold was an E-value filter of 1 × 10^−6^, default gradual EC weights, a GO weight of 5, and an annotation cutoff of 75. Un-annotated sequences were re-annotated with more permissive parameters. The sequences without BLAST hits and unannotated sequences were then processed on an InterProScan10 against EBI databases to retrieve functional annotations of protein motifs for merging with InterProScan GO terms. The GO annotation results were plotted using R scripts. The FASTA protein sequences of DEPs were blasted against the online KEGG database (http://geneontology.org/, accessed on 13 June 2024) to retrieve KOs for subsequent mapping to the KEGG11 pathways. The corresponding KEGG pathways were extracted.

GO enrichment at the three ontologies (biological process, molecular function, and cellular component) and KEGG pathway enrichment analyses were based on Fisher’s exact test, considering the whole quantified protein annotations as the background dataset. The Benjamini–Hochberg correction for multiple testing was applied to adjust the derived *p*-values, retaining only significant functional categories and pathways (with <0.05 *p*-values).

The relative protein expression data was used for hierarchical clustering analysis using Cluster3.0 (http://bonsai.hgc.jp/~mdehoon/software/cluster/software.htm, accessed on 13 June 2024) and the Java Tree view software (http://jtreeview.sourceforge.net, accessed on 13 June 2024). The Euclidean distance was used for similarity measure, average linkage (clustering uses the centroids of the observations), and hierarchical clustering. Finally, a heatmap was used to visualize the dendrogram. A PPI network analysis was performed by searching the string database (https://string-db.org/, accessed on 12 July 2024) and using the Cytoscape_3.7.2 software to explore interactions among DEPs [64].

### 4.7. Verification of DEPs by Parallel Reaction Monitoring (PRM) Analysis

The collection of xylem sap was based on a protocol that was Step 5.4, consistent with the samples for label-free-based proteomic analysis, three replicates were set up. The expression of the selected proteins was further quantified using LC-PRMMS analysis in Shanghai Applied Protein Technology Co., Ltd. (Shanghai, China). [65] to verify the protein expression levels obtained by label-free analysis. Briefly, peptides were prepared following the label-free protocol. Peptide Retention Time Calibration Mixture (Thermo Fisher Scientific, MA, USA) stable isotope peptides were spiked in each sample as the internal standard reference. Tryptic peptides were loaded on C18 stage tips for desalting before reversed-phase chromatography on an Easy nLC-1200 system (Thermo Fisher Scientific, MA, USA). One hour liquid chromatography gradients with acetonitrile ranging from 5 to 30% in 45 min were used. Then, PRM analysis was performed on a Q Exactive HF-X mass spectrometer (Thermo Fisher Scientific, MA, USA). Optimized methods for the collision energy, charge state, and retention times for the most significantly regulated peptides were generated experimentally using unique peptides of high intensity and confidence for each target protein. The mass spectrometer was operated in positive ion mode with the following parameters: full MS1 scan acquired with 60,000 resolution (300 *m*/*z*), 3.0 × 10^−6^ automatic gain control (ACG) target values, and 200 ms maximum ion injection times. Full MS scans were followed by 20 PRM scans at 30,000 resolution (at *m*/*z* 200) with AGC 3.0 × 10^−6^ and a maximum injection time of 100 ms. The targeted peptides were isolated with a 1.6 Th window. Ion activation/dissociation was performed at a normalized collision energy 27 in a higher energy dissociation (HCD) collision cell. The raw data was analyzed using Skyline (MacCoss Lab, University of Washington) [66], where signal intensities for individual peptide sequences for each significantly altered protein were quantified relative to each sample and normalized to a standard reference.

### 4.8. Quantitative Real-Time PCR (RT-qPCR) Analysis

An RT-qPCR was performed using the 11 genes selected based on the proteomic data. Table S3 provides the sequences for gene-specific primers designed using Primer Premier v5.0 [67]. As shown in Figure S1, the 2 cm stem part of the tobacco was cut as the raw material for the extraction of total RNA. Total RNA was extracted using the RNAiso Plus kit (TaKaRa Bio, Shiga, Japan), and first-strand cDNA synthesis was performed with the PrimeScript RT Reagent Kit (Takara, Dalian, China). Relative quantification was performed by RT-qPCR using the SuperReal PreMixPlus (SYBR Green) kit (Tiangen Biotech, Beijing, China) with Actin as the internal reference. Amplification efficiencies for all the genes were 95% to 110%, and all reactions were performed in triplicate. Relative gene expression was calculated using the 2^−ΔΔCt^ method [68]. All the data were statistically analyzed with GraphPad Prism (version 10.0). Statistical analysis included a one-way analysis of variance with Student’s *t*-test. Statistical significance was indicated as *p* < 0.05.

## 5. Conclusions

In this study, we observed TSWV virions in xylem vessels by TEM. Western blotting verified TSWV structural protein N and Gn presence in xylem sap. These results suggest that TSWV may engage in long-distance movement via the xylem. Further, proteomics was conducted to investigate the response of host plant xylem to TSWV infection. According to proteomic analysis, a total of 3305 proteins were identified in two groups. Among them, 315 host proteins (163 up-regulated and 152 down-regulated) and three viral structural proteins (N, Gn and Gc) were found differentially expression in two groups. GO enrichment analysis showed up-regulated proteins were significantly enriched in homeostasis, wounding, defense response, and DNA integration terms, while down-regulated proteins were significantly enriched in cell wall biogenesis/xyloglucan metabolic process-related terms. KEGG enrichment analysis showed that the differentially expressed proteins (DEPs) were most strongly associated with plant-pathogen interaction, the MAPK signaling pathway, and plant hormone signal transduction. Cluster analysis of DEPs function showed the DEPs can be categorized into cell wall metabolism-related proteins, antioxidant proteins, PCD-related proteins, host defense proteins such as receptor-like kinases (RLKs), salicylic acid binding protein (SABP), pathogenesis related proteins (PR), DNA methylation, and proteinase inhibitors (PIs). qRT-PCR and PRM assays illustrated the reliability of the label-free data and also suggested that proteins could be post-transcriptionally regulated. Our comparative proteomic analyses of *Nicotiana tabacum* xylem sap under TSWV stress help in understanding the host plant xylem system response to TSWV infection and provide an important basis for further study of the mechanism of TSWV long-distance movement in host plant vascular systems.

## Data Availability

Tables S1–S5.

## Supplementary Materials

The following supporting information can be downloaded at: https://www.mdpi.com/article/10.3390/ijms252010907/s1.
