# Supplementary material for: Integrated Biological Experiments and Proteomic Analyses of Nicotiana tabacum Xylem Sap Revealed the Host Response to Tomato Spotted Wilt Orthotospovirus Infection
Source: Int J Mol Sci. 2024 Oct 10;25(20):10907. doi: 10.3390/ijms252010907 (PMC11507450; doi:10.3390/ijms252010907)
Supplement: Supplementary file 1 [file ijms-25-10907-s001.zip › ijms-3210688-supplementary.pdf]

Table S1. Differentially expressed proteins for cluster analysis.

Protein ID	Gene ID	Protein description	P value	Up/Down
		Cell wall metabolism		
A0A1S3YNA4	LOC107777784	Probable polygalacturonase	0.022958865	down
A0A1S4D5M4	LOC107826279	Probable polygalacturonase	0.03346284	down
A0A1S3ZY07	LOC107791643	Xyloglucan endotransglucosylase/hydrolase	0.00511021	down
A0A1S4A648	LOC107794176	Xyloglucan endotransglucosylase/hydrolase	0.012514576	down
A0A1S3YM15	LOC107777521	glucan endo-1,3-beta-D-glucosidase	0.00733853	up
A0A1S4B2G1	LOC107803724	Xylose isomerase	0.04929654	up
A0A1S3YPW1	LOC107778308	Fasciclin-like arabinogalactan protein 12	0.014436925	down
A0A1S3XGA2	LOC107764751	Beta-xylosidase/alpha-L-arabinofuranosidase 2-like	0.000700394	down
		Protein kinase		
A0A1S4AG39	LOC107797310	phototropin-1-like	0.000001	down
A0A1S4DPS7	LOC107831858	UMP-CMP kinase	0.027525155	down
A0A1S3Y1Y5	LOC107771309	receptor-like protein kinase HERK 1	0.000001	down
A0A1S4C608	LOC107815464	probable receptor-like protein kinase At1g30570	0.000001	down
A0A1S4BXY1	LOC107813068	receptor-like protein kinase FERONIA	0.030151778	down
A0A1S3X1P1	LOC107771309	receptor-like protein kinase HERK 1	0.000001	down
A0A1S3XNF9	LOC107760318	somatic embryogenesis receptor kinase 2	0.000001	down
A0A1S4B8R9	LOC107805682	systemin receptor SR160-like	0.000001	down
A0A1S4CXR2	LOC107823752	SNF1-related protein kinase regulatory subunit gamma-1-like	0.000001	down
A0A1S4AQU6	LOC107800304	kinase-interacting family protein-like	0.032605722	down
A0A1S3ZKC5	LOC107787717	MOB kinase activator-like	0.000001	down
A0A1S3XH64	LOC107765190	somatic embryogenesis receptor kinase 2-like isoform X2	0.044246696	up
A0A1S4BF84	LOC107807623	cysteine-rich receptor-like protein kinase 10	0.000001	up
		Enzyme inhibitor		
Q03199	TIMPA	Proteinase inhibitor I-B	0.004418062	up
A0A1S4DG28	LOC107829494	Trypsin inhibitor 1-like	0.038967245	up
Q03198	TIMPB	Proteinase inhibitor(PI-IA)	0.014828036	up
A0A1S3XED7	LOC107764160	Cysteine proteinase inhibitor 5-like	0.005867287	up
A0A1S4BYK5	LOC107813255	Cysteine proteinase inhibitor	0.000639401	up
A0A1S3Z404	LOC107782545	21 kDa seed protein-like	0.001850605	up
Q3SBE6	AAZ20771.1	Insect injury-induced proteinase inhibitor	0.01357962	up
A0A097BU08	LOC107816051	miraculin-like	0.001723356	up
		Photosynthesis and chlorophyll metabolism		
A0A1S3XWY6	LOC107769513	PSI subunit V	0.035298994	down
A0A1S3ZIC0	LOC107787074	31 kDa ribonucleoprotein, chloroplastic-like	0.027259603	up
A9YTW5	LOC107825518	Putative red chlorophyll catabolite reductase	0.002839699	up
A0A1S4BPI1	LOC107810500	catechol oxidase	0.007182758	up
A0A1S4BB89	LOC107806492	Glucose-1-phosphate adenylyltransferase	0.049661681	up
		Transcription and regulator		
A0A1S4DQ97	LOC107832297	Glycine-rich protein 2-like	0.000353388	down
D6PZY5	RGP-1a	Glycine-rich RNA-binding protein-like（RGP-1a）	0.028122136	down
A0A1S4BSD9	LOC107811395	Heat shock factor-binding protein 1-like	0.040469993	down

Continue Table S1

Protein ID	Gene ID	Protein description	P value	Up/Down
		Transcription and regulator		
A0A1S4DPU2	LOC107831869	Uncharacterized oxidoreductase At4g09670-like	0.026552688	up
A0A1S3XPI1	LOC107767149	Ubiquitin-NEDD8-like protein RUB2	0.049935952	down
		Regulation of cellular process		
A0A1S3X2I0	LOC107760572	Thioredoxin Y1, chloroplastic-like	0.026018837	up
A0A1S3X5E0	LOC107761425	protein-disulfide reductase	0.001874837	up
A0A1S3YKW7	LOC107777121	Glutathione reductase	0.033974462	up
A0A1S4BKR7	LOC107809379	Protein disulfide-isomerase	0.017622643	up
A0A1S4CQA3	LOC107821493	protein-disulfide reductase	0.001153385	up
A0A1S4DPD3	LOC107831717	Protein disulfide-isomerase-like isoform X2	0.008963822	up
A0A1S4DPM9	LOC107832078	Protein disulfide-isomerase	0.008733416	up
		Response to stimulus and defense		
A0A1S3X398	LOC107760780	Copper transport protein ATX1-like isoform X2	0.007792509	down
A0A1S4AVA2	LOC107801743	Copper transport protein ATX1-like isoform X2	0.011433025	down
P23547	PR2	Glucan endo-1,3-beta-glucosidase, acidic isoform GI9（PR2）	0.007210626	up
P17514	XP_009788906.1	Acidic endochitinase Q	0.02575637	up
Q40558	PR-4A	Pathogenesis-related protein 4A（PR-4A）	9.64006E-05	up
A0A1S4BAT6	LOC107806372	Peroxidase	0.000532487	up
A0A1S3XQB7	LOC107767390	Peroxidase	0.048060327	up
Q50LG5	NtPOX1	Peroxidase	0.039070749	up
Q03199	TIMPA	Proteinase inhibitor I-B	0.004418062	up
A0A1S4DG28	LOC107829494	Trypsin inhibitor 1-like	0.038967245	up
Q03198	TIMPB	Proteinase inhibitor(PI-IA)	0.014828036	up
A0A1S4BKR7	LOC107809379	Protein disulfide-isomerase	0.017622643	up
A0A1S4BHB8	LOC107808276	Salicylic acid-binding protein 2-like	0.019369721	up
A0A1S3Y236	LOC107771200	acidic endochitinase P	0.015870874	up
P36401	NP_001412868.1	Glucan endo-1,3-beta-glucosidase, acidic isoform PR-Q'	0.008967076	up
A0A1S4DPM9	LOC107832078	Protein disulfide-isomerase	0.008733416	up
Q40597	CAA36790.1	Tobacco W38/1 PR-1 pathogenesis-related protein	0.010226347	up
Q53WU3	P08299.1	Tobacco PR-1a pathogenesis-related protein 1a	0.012922345	up
A0A1S3XH64	LOC107765190	Somatic embryogenesis receptor kinase 2-like isoform X2	0.023763894	up
A0A1S4CUX2	LOC107822846	Glutaredoxin	0.003318547	down
A0A1S4B1I2	LOC107803567	Superoxide dismutase	0.041151686	up
A0A1S4BHB3	LOC107808274	cytochrome c-like	0.000001	up
A0A1S4DJF0	LOC107830480	cathepsin B-like	0.000001	up
A0A1S4C9S4	LOC107816766	senescence-specific cysteine protease SAG12-like	0.000001	up
A0A1S3YCM0	LOC107774651	zingipain-2-like	0.045875785	up
P09042	LOC107763263	Pathogenesis-related protein 1C	0.000001	up
A0A1S3XXM2	LOC107769943	Pathogenesis-related protein R minor form	0.000001	up
A0A1S3XZH5	LOC107770326	Pathogenesis-related protein STH-2-like	0.000001	up
A0A1S4DJQ7	LOC107830465	Universal stress protein A-like protein	0.000001	up
P07053	D90197	Pathogenesis-related protein 1B	0.000001	up

Continue Table S1

Protein ID	Gene ID	Protein description	P value	Up/Down
Response to stimulus and defense				
P29060	LOC107825584	Acidic endochitinase	0.00222	up
Q7DM11	LOC107790755	Chitinase, class V	0.00634	up
P29061	LOC107807601	Basic endochitinase	0.000001	up
A0A1S4C0C3	LOC107813808	chitinase	0.00738	up
P23547	XM_009777473.1	Glucan endo-1,3-beta-glucosidase, acidic isoform GI9	0.00721	up
A0A1S3YM15	LOC107777521	glucan endo-1,3-beta-D-glucosidase	0.007338	up
A0A1S4CQI0	LOC107821387	Glucan endo-1,3-beta-glucosidase 7-like	0.02548	up
P36401	LOC107814850	Glucan endo-1,3-beta-glucosidase, acidic isoform PR-Q'	0.008967	up
B5APL3	EU867448.1	glucan endo-1,3-beta-D-glucosidase	0.04248	up
A0A1S4B7Z0	LOC107805507	Glucan endo-1,3-beta-glucosidase 5-like	0.04308	up
A0A1S3X2N3	LOC107760596	Glucan endo-1,3-beta-D-glucosidase-like isoform X2	0.04386	up
P52398	LOC107825406	Glucan endo-1,3-beta-glucosidase, acidic isoform GL161	0.000001	up
Sugar and energy metabolism				
A0A1S3Z7X6	LOC107783940	Alpha-amylase	0.02969394	up
P17514	XP_009788906.1	Acidic endochitinase Q	0.02575637	up
A0A1S3Y236	LOC107771200	acidic endochitinase P	0.015870874	up
P29060	NP_001313082.1	Acidic endochitinase	0.002228812	up
A0A1S4C0C3	LOC107813808	chitinase	0.007388001	up
B5APL3	ACF93731.1	glucan endo-1,3-beta-D-glucosidase	0.042489662	up
A0A1S3YM15	LOC107777521	glucan endo-1,3-beta-D-glucosidase	0.00733853	up
P23547	PR2	Glucan endo-1,3-beta-glucosidase, acidic isoform GI9（PR2）	0.007210626	up
P36401	NP_001412868.1	Glucan endo-1,3-beta-glucosidase, acidic isoform PR-Q'	0.008967076	up
A0A1S3X2N3	LOC107760596	Glucan endo-1,3-beta-D-glucosidase-like isoform X2	0.043867662	up
A0A1S4B7Z0	LOC107805507	Glucan endo-1,3-beta-glucosidase 5-like	0.043081818	up
A0A1S4CQI0	LOC107821387	Glucan endo-1,3-beta-glucosidase 7-like	0.02548792	up
Amino acid metabolism				
A0A1S4BKR7	LOC107809379	Protein disulfide-isomerase	0.017622643	up
A0A1S4D1P6	LOC107824997	Zingipain-1-like	0.020636425	up
A0A1S4DPM9	LOC107832078	Protein disulfide-isomerase	0.008733416	up
A0A1S3XED7	LOC107764160	Cysteine proteinase inhibitor 5-like	0.005867287	up
A0A1S3YCM0	LOC107774651	Zingipain-2-like	0.010708522	up
A0A1S4DG28	LOC107829494	Trypsin inhibitor 1-like	0.038967245	up
A0A1S3Z4W9	LOC107782952	Glutathione S-transferase	0.04820231	up
A0A1S4C8H1	LOC107816353	glutathione transferase	0.032986128	up
Q03199	TIMPA	Proteinase inhibitor I-B	0.004418062	up
A0A1S3X2I0	LOC107760572	Thioredoxin Y1, chloroplastic-like	0.026018837	up
Q3SBE6	AAZ20771.1	Insect injury-induced proteinase inhibitor	0.01357962	up
A0A1S3XA26	LOC107762876	cysteine desulfurase	0.009550189	up
A0A1S4ASD7	LOC107800866	glutathione transferase	0.006705954	up
A0A1S4BSF5	LOC107811393	Uncharacterized protein LOC107811393 isoform X1	0.002305696	up
A0A1S4C1Y2	LOC107814292	glutathione transferase	0.023504634	up

Continue Table S1

Protein ID	Gene ID	Protein description	P value	Up/Down
		Amino acid metabolism		
A0A1S4BYK5	LOC107813255	Cysteine proteinase inhibitor	0.000639401	up
A0A1S3XNA9	LOC107767031	Uncharacterized protein LOC107767031	0.005589462	up
A0A1S4BXY1	LOC107813068	Receptor-like protein kinase FERONIA	0.020656895	down
A0A1S3ZFS6	LOC107786350	Xylem cysteine proteinase 1-like	0.027035416	down
A0A1S4CPL5	LOC107821114	Profilin	0.002092124	down
A0A1S3XPI1	LOC107767149	Ubiquitin-NEDD8-like protein RUB2	0.049935952	down
A0A1S4DGT5	LOC107829640	DnaJ protein homolog	0.018014843	down
A0A1S4CUX2	LOC107822846	Glutaredoxin	0.003318547	down
A0A1S4C929	LOC107816410	Proteasome subunit alpha type	0.001065787	up
A0A1S4D5K0	LOC107826219	Isovaleryl-CoA dehydrogenase, mitochondrial	0.009771046	up
A0A1S4BNK8	LOC107810220	putative methyltransferase DDB_G0268948	0.000001	up
A0A1S3WXP0	LOC107759094	Serine hydroxymethyltransferase	0.000001	up
P68173	SAHH	Adenosylhomocysteinase	0.046164558	up
		Cofactor binding		
A0A1S3X0N1	LOC107759965	Gamma aminobutyrate transaminase 3, chloroplastic-like	0.023504634	up
A0A1S3XA26	LOC107762876	cysteine desulfurase	0.009550189	up
A0A1S4AZA4	LOC107802933	Gamma aminobutyrate transaminase 1, mitochondrial isoform X1	0.039644263	up
A0A1S4CPB5	LOC107821173	Gamma aminobutyrate transaminase 3, chloroplastic isoform X2	0.039503406	up
A0A1S3YKW7	LOC107777121	Glutathione reductase	0.033974462	up
A0A1S4D4R1	LOC107826001	2-methylacyl-CoA dehydrogenase, mitochondrial-like isoform X2	0.026177536	up
A0A1S4D5K0	LOC107826219	Isovaleryl-CoA dehydrogenase, mitochondrial	0.009771046	up
A0A1S4BB89	LOC107806492	Glucose-1-phosphate adenylyltransferase	0.049661681	up
A0A1S4DPB8	LOC107831711	Persulfide dioxygenase ETHE1 homolog, mitochondrial-like	0.046299242	up
Q9FXS7	EIG-I24	EIG-I24 protein（EIG-I24）	0.020007316	up
A0A1S4DRQ6	LOC107832751	4-hydroxyphenylpyruvate dioxygenase	0.025177063	up
A0A1S4D817	LOC107826954	Acyl-CoA-binding protein-like	0.045984256	down

Note: *P* value =0.000001 indicates that the protein is present only in the infected-group or control-group.

Table S2. Differentially expressed proteins for PPI interaction network analysis

Protein ID	Gene ID	Protein description	Up/Down
a.Carbohydrate metabolic process and defense response			
P68158	TUFA	Elongation factor Tu, chloroplastic	down
A0A1S4DRQ6	LOC107832751	4-hydroxyphenylpyruvate dioxygenase	up
A0A1S4BHB3	LOC107808274	Cytochrome c-like	up
A0A1S4AZJ1	LOC107802918	Dihydrolipoyl dehydrogenase 2, chloroplastic-like	up
A0A1S3WXP0	LOC107759094	Serine hydroxymethyltransferase	up
A0A1S4D5K0	LOC107826219	Isovaleryl-CoA dehydrogenase, mitochondrial	up
A0A1S4BBF5	LOC107806500	Triosephosphate isomerase, chloroplastic-like	up
A0A1S3YTS3	LOC107779604	phosphopyruvate hydratase	down
A0A1S3Z7X6	LOC107783940	Alpha-amylase	up
A0A1S4DNT5	LOC107831547	Pyruvate dehydrogenase E1 component subunit beta	up
A0A1S3Y236	LOC107771200	acidic endochitinase P	up
A0A1S4ASI2	LOC107800746	Uncharacterized protein LOC107800746	up
A0A1S3XUT0	LOC107768957	Cytochrome b5-like	down
P17514	XP_009788906.1	Acidic endochitinase Q	up
A0A1S4AJS1	LOC107798454	beta-fructofuranosidase	down
A0A1S3ZIE8	LOC107787145	Acyl-CoA-binding protein	down
A0A1S4D817	LOC107826954	Acyl-CoA-binding protein-like	down
A0A1S4AEZ3	LOC107796952	Eukaryotic translation initiation factor 5B	down
A0A1S3XA26	LOC107762876	cysteine desulfurase	up
Q43576	chi-V	Chitinase, class V	up
A0A1S4DPS7	LOC107831858	UMP-CMP kinase	down
A0A1S3ZK76	LOC107787846	3-dehydroquinate synthase	up
A0A1S3Z9P3	LOC107784541	fructose-bisphosphatase	down
A0A1S4DPB8	LOC107831711	Persulfide dioxygenase ETHE1 homolog, mitochondrial-like	up
A0A1S3Z5N9	LOC107783336	Protein DJ-1 homolog D-like	up
A0A1S4B2G1	LOC107803724	Xylose isomerase	up
P09042	NP_001412683.1	Pathogenesis-related protein 1C	up
P29060	NP_001313082.1	Acidic endochitinase	up
P23547	PR2	Glucan endo-1,3-beta-glucosidase, acidic isoform GI9	up
A0A1S4AK94	LOC107798617	Pathogenesis-related leaf protein 6-like	down
O50038	SCANT	Secoisolariciresinol dehydrogenase-like	up
A0A1S4BNB6	LOC107810157	Protein BOLA2-like	down
A0A1S3X2I0	LOC107760572	Thioredoxin Y1, chloroplastic-like	up
A0A1S4BB89	LOC107806492	Glucose-1-phosphate adenylyltransferase	up
A0A1S3X559	LOC107761175	Adenosylhomocysteinase	up
A0A1S4DQ97	LOC107832297	Glycine-rich protein 2-like	down
A0A1S4D2P6	LOC107825356	Uncharacterized protein LOC107825356	up
A0A1S4CVQ7	LOC107823113	Cytochrome b5-like	up
A0A1S4CUX2	LOC107822846	Glutaredoxin	down
A0A1S4BNE9	LOC107810166	Glycine-rich protein 2-like	up
A0A1S4DJF0	LOC107830480	Cathepsin B-like	up

Continue Table S2

Protein ID	Gene ID	Protein description	Up/Down
a.Carbohydrate metabolic process and defense response			
A0A1S4DJQ7	LOC107830465	Universal stress protein A-like protein	up
A0A1S4DJF0	LOC107830480	Cathepsin B-like	up
A0A1S4DB47	LOC107827955	ADP/ATP translocase	up
A0A1S4BBV8	LOC107806708	ATP-dependent Clp protease proteolytic subunit	up
A0A1S4BKR7	LOC107809379	Protein disulfide-isomerase	up
A0A1S4DPM9	LOC107832078	Protein disulfide-isomerase	up
A0A1S4AVT9	LOC107801938	Mitochondrial-processing peptidase subunit alpha-like	down
A0A1S4BF12	LOC107807586	Glucan endo-1,3-beta-glucosidase 6-like	down
A0A1S4B7Z0	LOC107805507	Glucan endo-1,3-beta-glucosidase 5-like	up
A0A1S4AJI1	LOC107798126	Probable Xaa-Pro aminopeptidase P	up
A0A1S4A7I0	LOC107794569	Uncharacterized protein LOC107794569	up
A0A1S3ZTG2	LOC107790262	Eukaryotic peptide chain release factor GTP-binding subunit	down
A0A1S3ZPM9	LOC107789090	Short-chain type dehydrogenase/reductase-like	up
A0A1S4DGM7	LOC107829691	Miraculin-like	up
A0A1S4C4M9	LOC107815033	Clathrin heavy chain 1-like	up
A0A1S3Z6Q6	LOC107783614	Uncharacterized protein LOC107783614	up
A0A1S4B1I2	LOC107803567	Superoxide dismutase	up
A0A1S3Z2N1	LOC107782091	Universal stress protein A-like protein	down
A0A1S3XGW1	LOC107765053	glucose-6-phosphate 1-epimerase	down
A0A1S3WY43	LOC107759237	H/ACA ribonucleoprotein complex subunit 4-like	down
b.Cellular response to heat,protein folding and protein stabilization			
A0A1S3XL99	LOC107766483	Heat shock 70 kDa protein 15-like	down
A0A1S4CCK2	LOC107817520	Heat shock protein 83-like	up
A0A1S4AHN5	LOC107797756	Chloroplastic heat shock protein 90	down
A0A1S4BSD9	LOC107811395	Heat shock factor-binding protein 1-like	down
A0A1S3ZX95	LOC107791417	Uncharacterized protein LOC107791417	down
A0A1S3Z024	LOC107781579	DnaJ homolog subfamily B member 4-like	down
A0A1S4DGT5	LOC107829640	DnaJ protein homolog	down
A0A1S3ZIF8	LOC107787048	3-hexulose-6-phosphate isomerase-like	down
c.Proteasomal protein catabolic process, ubiquitin-dependent protein catabolic process and chlorophy II catabolic process			
A0A1S3XPI1	LOC107767149	Ubiquitin-NEDD8-like protein RUB2	down
A0A1S4BL45	LOC107809483	Ubiquitin receptor RAD23c-like isoform X1	down
A0A1S3Z4U1	LOC107782966	Proteasome subunit alpha type	up
A0A1S4C929	LOC107816410	Proteasome subunit alpha type	up
A0A1S4DRG6	LOC107832680	NEDD8 ultimate buster 1-like	up
A9YTW5	LOC107825518	Putative red chlorophyll catabolite reductase	up
A0A1S4BLS6	LOC107809646	Uncharacterized protein LOC107809646 isoform X1	down
d.Microtubule cytoskeleton organization and signal transduction			
Q549Z2	Nt14-3-3omega3	14-3-3 protein	up

Continue Table S2

Protein ID	Gene ID	Protein description	Up/Down
d.Microtubule cytoskeleton organization and signal transduction			
A0A1S4DDE4	LOC107828441	Vacuolar protein sorting-associated protein 32 homolog 2-like	down
Q8VXD0	tubA2	Tubulin alpha chain	down
A0A1S4B8R9	LOC107805682	non-specific serine/threonine protein kinase	down
A0A1S4AG39	LOC107797310	non-specific serine/threonine protein kinase	down
A0A1S3X1P1	LOC107760318	non-specific serine/threonine protein kinase	down
e.Photosynthesis			
P27493	CAB21	Chlorophyll a-b binding protein 21, chloroplastic	down
A0A1S3XCI2	LOC1077636388	Rab escort protein 1	down
A0A1S3YAU9	LOC107774251	Chlorophyll a-b binding protein, chloroplastic	down
Q84QE7	LOC107789388	Photosystem I reaction center subunit III	up
A0A1S3XWY6	LOC107769513	PSI subunit V	down
f.Plant-type secondary cell wall biogenesis			
A0A1S4BS33	LOC107811311	14 kDa zinc-binding protein-like	up
A0A1S4BXM6	LOC107812975	Fasciclin-like arabinogalactan protein 11	down
A0A1S4C4L3	LOC107815072	DNA-directed RNA polymerases II and V subunit 8A-like	down
A0A1S4BJ43	LOC107808859	Probable 3-hydroxyisobutyrate dehydrogenase-like 1, mitochondrial	up
Rests			
A0A1S4D404	LOC107825693	Abscisic acid and environmental stress-inducible protein TAS14-like	down
A0A1S4DB48	LOC107827875	Non-specific lipid-transfer protein	down
A0A1S4CN99	LOC107820710	Non-specific lipid-transfer protein	up
A0A1S4CI96	LOC107819142	Thioredoxin-like protein YLS8	down
A0A1S4DKS1	LOC107830830	UBP1-associated protein 2B-like	down
A0A1S3YQM5	LOC107778732	U2 small nuclear ribonucleoprotein A'-like	down
A0A1S4CPL5	LOC107821114	Profilin	down
A0A1S4ATS9	LOC107801297	Profilin	down
A0A1S4ABD2	LOC107795742	Peamaclein-like	down
A0A1S3YCK4	LOC107774806	Thaumatin-like protein	down
A0A1S3XZH5	LOC107770326	Pathogenesis-related protein STH-2-like	up
Q03199	TIMPA	Proteinase inhibitor I-B	up
A0A1S3Z5U0	LOC107783309	Oxalate--CoA ligase-like	up
A0A1S4D1N7	LOC107824883	catechol oxidase	down
A0A1S4BPI1	LOC107810500	catechol oxidase	down
D6PZY5	RGP-1a	Glycine-rich RNA-binding protein-like	down
A0A1S4BYK5	LOC107813255	Cysteine proteinase inhibitor	up

Table S3. Protein description for qRT-PCR

Protein ID	Gene ID	Protein description	Abbreviations
A0A1S3Y1F7	LOC107827955	probable ADP,ATP carrier protein At5g56450	CP450
A0A1S4B7Z0	LOC107805507	Glucan endo-1,3-beta-glucosidase 5-like	GL153
A0A1S4BKR7	LOC107809379	Protein disulfide-isomerase	PDI
A0A1S3YCM0	LOC107774651	Zingipain-2-like	Zingipain-2-like
A0A1S4DJF0	LOC107830480	Cathepsin B-like	Cathepsin B
Q9ZSD5	LOC107790139	Syntaxin-related protein Nt-syr1	Nt-syr1
A0A1S4DGB9	LOC107829474	Trypsin inhibitor 1	T1
A0A1S4C608	LOC107815464	probable receptor-like protein kinase At1g30570	RLK-AtIg30570
A0A1S4CPL5	LOC107821114	Profilin	Profilin
A0A1S3YNA4	LOC107777784	probable polygalacturonase	PG
A0A1S4DQ97	LOC107832297	glycine-rich protein 2-like	GRP

Table S4. Primer sequences for qRT-PCR

Primer name	Primer sequence(5′-3′)
actin-F	CAAGGAAATCACCGCTTTGG
actin-R	AAGGGATGCGAGGATGGA
CP450-F	AGGATGGTGAGGGTGAACAG
CP450-R	TGCAATCAAACATGCCCTTA
GL153-F	ATAGCCAATGGTTGGGTTCA
GL153-R	TAGCTTTGGGTGGGTAGGTG
PDI-F	CTCGTGAGACAGACGGTATAATC
PDI-R	GCTTGCAGCATCTTCCTTTG
zingipain-2-like-F	GAGTGGTGCTGTCACTAGAATC
zingipain-2-like-R	GTTGAGAGTTTGTTTGCTCCTTC
Cathepsin B-F	ATCGGATGGGGAACC
Cathepsin B-R	TTTGGCTGAAGGCAAT
Nt-syr1-F	CGAGCTAAGGCAAAAGATGG
Nt-syr1-R	GCTCTTGAATTGCCTTTTGC
T1-F	GTGAAGTTAGCTCTCGTGGTTAT
T1-R	GTTCTGGCCATGTCTCCTTT
RLK-AtIg30570-F	CATCTCTTTGGCAGCGATCTA
RLK-AtIg30570-R	CTCTGACCCTGTATGAAGGTAATG
profilin-1-F	TCACCATGACTGCCAAGAC
profilin-1-R	CAGGTGAGAAGCCATTTCATAAC
PG-F	GGGCAGTTATAGACCCATTACC
PG-R	GCCCATCTATTGTCCCATTCT
GRP-F	AGGTGGGAGATATGGTGGTAG
GRP-R	CACTGCTGTTAGGGCATTCA

Table S5. Comparison of the quantification results between Label-free and PRM of the 20 candidate proteins

Protein ID	Gene ID	Protein description	Abbreviations	Label-free result	PRM result
				TSWV/NC[a]	TSWV/NC[a]
A0A1S3ZFS6	LOC107786350	Xylem cysteine proteinase 1-like	XCP1	0.305302207	0.365798295
A0A1S3XJ20	LOC107765742	miraculin-like	miraculin-like	0.348470233	0.726357106
A0A1S3YNA4	LOC107777784	Probable polygalacturonase	PG	0.460295739	0.80211781
A0A1S3XED7	LOC107764160	Cysteine proteinase inhibitor 5-like	CPI	3.783538454	2.568875084
A0A1S3X2N3	LOC107760596	Glucan endo-1,3-beta-D-glucosidase-like isoform X2	EGLC	2.666492637	4.056962686
A0A1S3XA26	LOC107762876	cysteine desulfurase	cd	5.874029152	4.246276256
A0A1S4DRQ6	LOC107832751	4-hydroxyphenylpyruvate dioxygenase	HPPD	2.464053596	5.101581409
A0A1S4BKR7	LOC107809379	Protein disulfide-isomerase	PDI	2.582862671	5.54353316
A0A1S4BHB8	LOC107808276	Salicylic acid-binding protein 2-like	SABP2	2.96356218	5.910116807
A0A1S4B1I2	LOC107803567	Superoxide dismutase	SOD	3.66449449	6.155615534
Q43576	LOC107790755	Chitinase, class V	chi-V	4.41637032	6.358114729
A0A1S3ZPM9	LOC107789090	Short-chain type dehydrogenase/reductase-like	SDR	2.248793793	6.695955674
Q40575	AAA34121.1	SAR8.2e protein	SAR8.2e	2.042164868	9.323050458
Q9FXS7	LOC107773785	EIG-I24 protein（EIG-I24）	EIG-I24	3.919272686	10.36385849
Q40558	LOC107802320	Pathogenesis-related protein 4A（PR-4A）	PR-4A	9.099695837	15.80287112
A0A1S4BAT6	LOC107806372	Peroxidase	POD	6.335924954	30.95421624
A0A1S4CQA3	LOC107821493	protein-disulfide reductase	PDR	8.223255197	42.78245611
P17514	XM_009790604.1	Acidic endochitinase Q	PR-Q	10.38666232	78.2180688
P23547	XM_009777473.1	Glucan endo-1,3-beta-glucosidase, acidic isoform GI9（PR2）	PR2	30.77275519	240.4231559
A0A1S4D1P6	LOC107824997	Zingipain-1-like	Zingipain-1-like	115.0621714	372.8469396

Note: [a] Fold changes of protein abundances between TSWV-infection and control in tobacco xylem sap.

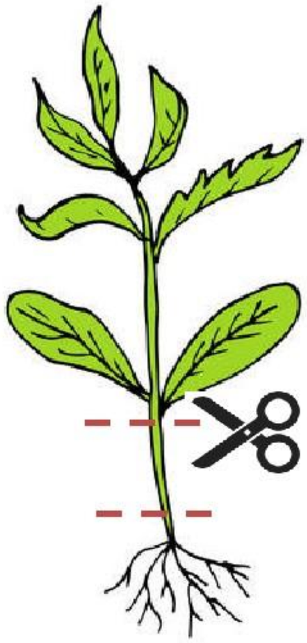

Figure S1. The xylem sap collection procedure.
